# Mechanisms of Entry and Endosomal Pathway of African Swine Fever Virus

**DOI:** 10.3390/vaccines5040042

**Published:** 2017-11-08

**Authors:** Elena G. Sánchez, Daniel Pérez-Núñez, Yolanda Revilla

**Affiliations:** Virology Department, Centro de Biología Molecular Severo Ochoa, Consejo Superior de Investigaciones Científicas-Universidad Autónoma de Madrid (CSIC-UAM), 28049 Madrid, Spain; elena_garcia@cbm.csic.es (E.G.S.); daniel_perez@cbm.csic.es (D.P.-N.)

**Keywords:** ASFV, viral entry, macropinocytosis, CME, endosomes, signaling, PI3K, Rac-1, Pak-1, macrophages, uncoating

## Abstract

African Swine Fever Virus (ASFV) causes a serious swine disease that is endemic in Africa and Sardinia and presently spreading in Russia and neighboring countries, including Poland and recently, the Czech Republic. This uncontrolled dissemination is a world-wide threat, as no specific protection or vaccine is available. ASFV is a very complex icosahedral, enveloped virus about 200 nm in diameter, which infects several members of pigs. The virus enters host cells by receptor-mediated endocytosis that depends on energy, vacuolar pH and temperature. The specific receptor(s) and attachment factor(s) involved in viral entry are still unknown, although macropinocytosis and clathrin-dependent mechanisms have been proposed. After internalization, ASFV traffics through the endolysosomal system. The capsid and inner envelope are found in early endosomes or macropinosomes early after infection, colocalizing with EEA1 and Rab5, while at later times they co-localize with markers of late endosomes and lysosomes, such as Rab7 or Lamp 1. A direct relationship has been established between the maturity of the endosomal pathway and the progression of infection in the cell. Finally, ASFV uncoating first involves the loss of the outer capsid layers, and later fusion of the inner membrane with endosomes, releasing the nude core into the cytosol.

## 1. Mechanisms of ASFV Entry

Endocytosis, or internalization through endosomes, is an efficient mechanism used by many viruses to overcome the physical barrier of the cellular plasma membrane, entering the cell to initiate productive infection. The type of endocytosis used depends on the activation of specific cell signaling pathways, driven by virus-cell interactions. Knowledge of the signaling pathways that trigger viral entry and related mechanisms is central to the understanding of host-cell interactions and viral pathogenesis.

African Swine Fever Virus (ASFV) is one such virus that enters the host cell by endocytosis, a process first observed in transmission electron microscopy (TEM) studies that showed viral particles internalizing in cytoplasmic vesicles from the cell membrane; besides, the infection was inhibited in the presence of lysosomotropic drugs that increase vacuolar pH [1,2,3]. The cellular and viral factors involved in this process as well as the different endocytosis mechanisms used by ASFV to enter host cells are discussed in the present review.

### 1.1. Cellular Receptors and Viral Proteins Involved in ASFV Entry

Currently the identity of the cellular receptors involved in viral entry are still unknown, although mediation of ASFV entry by saturable receptors present on the cellular plasma membrane has been described in porcine macrophages and Vero cells [4,5]. Nevertheless, the existence of these receptors is necessary but not sufficient for efficient viral production, as they are not the only factors affecting productive infection [6]. These studies demonstrated that in some cases, i.e., in rabbit macrophages, the virus is able to bind to the plasma membrane by a non-saturable binding, allowing viral internalization and synthesis of some early viral proteins, although the infection is abortive in these cells [4,5].

Regarding viral entry in porcine alveolar macrophages (PAM), the natural target cell of the virus in the animal, it has been claimed that infection does not occur via Fc receptors, and, therefore, that antibody-dependent viral entry does not assist viral infection [7]. However, the role of Fc receptors in ASFV entry is still under discussion based on a study in which DNA vaccines induced a strong humoral response but exacerbated viremia in pigs [8]. Similar results were obtained in a separate study in which a combination of ASFV DNA and recombinant viral proteins also induced an antibody response that presumably enhanced infection in vivo [9]. On the other hand, CD163, a member of the scavenger receptor cysteine-rich domain family whose expression is restricted to cells of the monocyte/macrophage lineage, has been postulated to be important for viral entry, since ASFV infection is enhanced after the in vitro maturation of porcine blood monocyte cells (PBMCs) to macrophages (which correlates to CD163 up-regulation). A higher number of ASFV-infected cells were found in magnetically-fractionated PBMCs expressing CD163 than in those lacking the receptor. Moreover, when macrophages were incubated with a specific anti-CD163 antibody, the levels of infection and viral binding decreased in a dose-dependent manner [10]. However, the role of CD163 in ASFV infection remains controversial since it was recently published that in non-permissive cells, CD163 expression is not enough to increase susceptibility to ASFV [11], and that CD163-knockdown pigs are not resistant to infection with ASFV Georgia 2007/1 [12]. These observations are in line with a recent study where NHV/P68-ASFV was efficiently produced after the infection of WSL cells despite only about 6% of the cells being positive for CD163, indicating that this receptor is not essential for ASFV infection in several cases [13].

On the other hand, characterization of the viral ligand has been the subject of great attention, and currently viral protein p12 has been described as the factor (or one of the factors) responsible for ASFV binding to host cells [14]. Infection was blocked in vitro when cells were incubated with recombinant p12 protein, though specific anti-p12 antibodies were not able to fully inhibit virus binding or neutralize viral infectivity, suggesting that it is not the only factor responsible for viral adsorption to cells [15]. The involvement of viral proteins p32, p72 and p54 has been described in the early stages (binding/internalization) of the infectious cycle in both Vero cells and swine macrophages [16,17]. These studies reported that infection was efficiently inhibited in vitro when cells were incubated with sera from convalescent pigs infected with attenuated E75CV1-4. Furthermore, pig sera obtained after the inoculation of pigs with recombinant p72, p54 and p32 proteins showed the role of both p72 and p54 in viral binding and the role of p32 in viral internalization [17]. When these recombinant proteins were used to assess the neutralization of ASFV E75 infection in pigs, only partial protection was obtained with the combination of p54 and p32 recombinant proteins [16]. Similarly, another study reported that the combination of baculovirus-expressed p32, p54, p72 and p22 proteins were not able to protect against the virulent isolate Pr4. Altogether, these results suggest that other viral factors apart from p72, p32 and p54 must be involved in viral entry. Current studies have shown that sera from pigs inoculated with a combination of several structural and non-structural ASFV proteins together with viral DNA partially neutralized infection when incubated with sera from both virulent and attenuated ASFV strains [9].Therefore, further studies are needed to accurately define both the cellular and viral factors involved in the first steps of ASFV infection.

### 1.2. Viral Internalization: Endocytosis

#### 1.2.1. ASFV Entry Depends on Temperature, Cholesterol, Energy and Vacuolar pH 

After binding to the cellular plasma membrane, ASFV enters host cell by endocytosis. Unlike other viral systems such as HIV (Human Immunodeficiency Virus), which uses both endocytosis and direct fusion to enter cells [18,19], ASFV requires only endocytosis to initiate productive infection. TEM studies have shown that fusion of the cellular and viral membranes can be artificially induced by decreasing the pH, though infection does not progress if it occurs in the presence of lysosomotropic drugs, suggesting that virions forced to internalize by a process other than endocytosis are degraded in the cytoplasm [3,20,21]. Endocytosis is dependent on temperature and energy. The percentage of viral particles attached to the cellular membrane after 3 h of incubation in Vero cells is double at 37 °C than at 4 °C after 3 h of virus incubation in Vero cells; moreover, the internalization is completely blocked in the presence of metabolic inhibitors or incubation at 4 °C [1,21]. Finally, during ASFV, endocytosis cholesterol plays an important role since it is key to the fusion between the viral and endosomal membranes [22].

#### 1.2.2. Endocytic Pathways Used by ASFV: CME and Macropinocytosis

**Figure 1:**
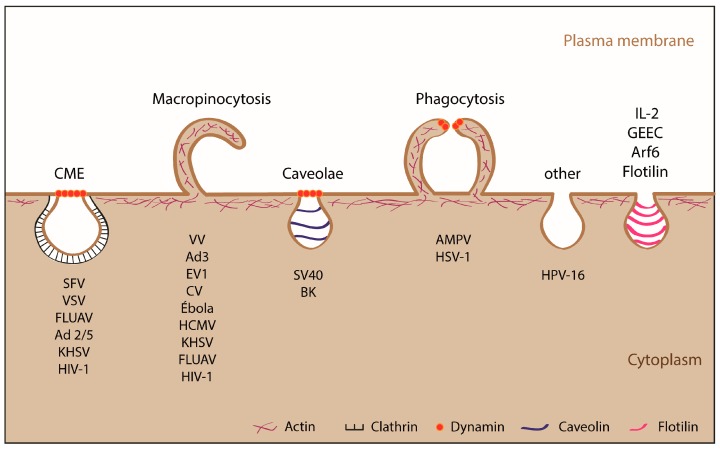
Endocytic mechanisms used by animal viruses. In general viruses smaller than 150 nm internalize by clathrin-mediated endocytosis (CME) or caveolae mechanisms, whereas those larger than 150–200 nm use macropinocytosis or phagocytosis. Other mechanisms such as Arf-6, IL-2, flotilin and GPI-AP enriched early endosomal compartment (GEEG) are less commonly used by viruses to enter into cell. It is important to note that some viruses can enter the cell through more than one type of endocytosis. SFV, Semliki Forest Virus; VSV, Vesicular Stomatitis Virus; FLUAV, Influenza A Virus; Ad2/5, Adenovirus 2 and 5; KHSV, Kaposi Sarcoma-associated Herpesvirus; HIV-1, Human Immunodeficiency Virus; VV, Vaccinia Virus; Ad3, Adenovirus 3; EV1, Echovirus 1; CV, Coxsackie Virus, HCMV, Human Cytomegalovirus; SV40, Simian Vacuolating Virus 40; BK, Virus BK; AMPV, Mimivirus; HSV-1, Herpes Simplex Virus; HPV-16, Human Papillomavirus 16. Adapted from [36].

In recent years, great interest has emerged in defining the endocytic mechanism used by ASFV for entry into the host cell. As a result of several studies, it has been reported that the virus is able to internalize via two distinct endocytic pathways: macropinocytosis and clathrin-mediated endocytosis (CME). This versatility in host-cell entry, also seen in other viruses [23,24,25,26], increases the odds of success by activating different signaling pathways to overcome the barriers the cell uses to prevent viral infection. Viruses have been shown to enter cells using many endocytosis-related pathways, including macropinocytosis, CME, caveolae, phagocytosis and others (Figure 1).

CME is probably the best characterized mechanism for viral entry into cells [27]. It is a rapid and efficient process that occurs constitutively in all mammalian cells to internalize nutrients, growth factors, antigens, pathogens and plasma membrane receptors [28]. During CME, viral particles internalize with their specific receptors via coated membrane invaginations (coated pits), which rapidly form coated vesicles [29] of between 85–120 nm diameter. The main component of these invaginations and vesicles is clathrin [30,31,32], which is recruited to the membrane by the AP-2 adapter complex with participation of other accessory molecules such as AP180, Eps15, amphiphysin, endophylline and phosphatidylinositols [32]. Finally, this vesicle is cleaved from the plasma membrane by the GTPase dynamin [33,34,35]. Once in the cytoplasm, the vesicles lose clathrin and give rise to early endosomes that continue their maturation process [29]. 

Due to the restrictions imposed by the large size of the ASFV viral particle (200 nm), CME was not initially considered to be the most probable mechanism for viral entry, as it is more commonly used by smaller viruses such as the Adenovirus 2/5 [26,37] and Semliki Forest Virus [38]. Nevertheless, TEM studies have detected ASFV viral particles within dense, possibly clathrin-coated membrane invaginations and vesicles in Vero cells infected with the Ba71V isolate, and in swine alveolar macrophages infected with the E70 isolate [1,39]. Moreover, it has been reported that expression of the early viral protein p32 is inhibited by the expression of Eps15 and dynamin-2 dominant negative in Vero cells infected with Ba71V [40]. In that study, about 60% of the viral particles colocalized with the heavy chain of clathrin during the first 20 min of internalization, and the expression of p32 was inhibited when the cells were treated with dynasore (specific inhibitor of dynamin) and chlorpromazine (CPZ; CME inhibitor). In contrast, another study showed that in the same experimental model, viral uptake was only partially reduced with dynasore treatment but not with CPZ, although the expression of viral proteins was inhibited by both treatments [41], suggesting that CPZ may affect later stages of viral entry. These controversial data may possibly be explained by differences in the experimental approaches used in these two studies. Further clarification was obtained from two studies reporting that different viral isolates, such as Ba71V, 608V13 and E70, use CME to internalize into porcine alveolar macrophages [39,42]. While ASFV uptake was reduced in a dose-dependent manner after treatment with specific inhibitors of CME, including dynasore, CPZ and pitstop2, the authors could not rule out that some of these treatments have side effects that may have affected other viral processes. Moreover, 25% of the viral particles were localized to vesicles along with transferrin, a specific fluid phase marker of CME, and the expression of early viral protein p32 was compromised when PAM were transfected with siRNAs specific for the clathrin-heavy chain [39], supporting that CME has some role in virus entry.

Macropinocytosis is an actin-dependent process associated with dynamic plasma membrane activity, forming protrusions (ruffles or blebs) triggered by the activation of receptors and kinases such as Pak-1, PI3K, EGFR and Rho GTPases. Using this endocytic pathway, viruses internalize into large (0.5–10 mm) uncoated vesicles called macropinosomes [43,44]. This route is a constitutive process in macrophages, although treatment with epithelial growth factor (EGF), macrophage-colony stimulating factor (rM-CSF) or phorbol 12- myristate 13- acetate (PMA) enhances it significantly [45,46,47,48]. As a consequence of this stimulation, some cellular tyrosine kinase receptors are also activated, inducing changes in the actin cytoskeleton and generating the characteristic membrane protrusions. 

In relation to macropinocytosis, a recent report has demonstrated that ASFV internalizes efficiently by this mechanism in PAM [39]. The virus was found in vesicles as macropinosomes containing dextran, a specific marker that internalizes by this mechanism. Moreover, when cells were treated with different specific inhibitors of macropinocytosis such as EIPA (Na^+^/H^+^ channel inhibitor), IPA-3 (Pak-1 inhibitor) or Cytochalasin D (inhibitor of actin filament polymerization), both viral uptake and p32 expression was compromised. Viral internalization was completely inhibited when the infection was carried out in the presence of both EIPA and CPZ, reaffirming the importance of the two endocytic pathways in porcine alveolar macrophages. The authors claimed that since macropinocytosis is constitutively activated in macrophages, the “ruffling” or “blebbing” was not specifically induced by the virus, although viral particles were detected between the existing membrane protrusions to be engulfed. These results suggest that, even though ASFV uses macropinocytosis to enter into porcine alveolar macrophages, this endocytic pathway is not induced by the virus in macrophages, partially supporting the observed lack of Pak-1 phosphorylation [39]. 

On the other hand, it is important to note that a primary culture of alveolar macrophages displays a major degree of heterogeneity, and the type or the degree of activation of the ASFV-infected cells has not been approached. In this regard, it is known that macrophages are able to undergo maturation towards a pro- (M1) or anti-inflammatory (M2) phenotype in response to different stimuli, including certain viruses, such as HIV [49]. These processes are related to the expression of several cellular receptors and patterns of cytokine secretion [50,51]. Recently it has been published that replication of ASFV in M1 or M2 subpopulations depends on the virulence of the strain, since the virulent 22653/14 displayed a higher capacity to infect M1 macrophages than the Vero-adapted strain Ba71V. This suggests that virulent strains have developed mechanisms to more effectively counteract macrophage activation, to ensure their dissemination through the host [52]. The functional role of M1 or M2 macrophages is related to their susceptibility to productive infection by ASFV and has also been recently approached by our laboratory [53]. More accurate studies describing the differential entry process of attenuated and virulent ASFV into M1 or M2 macrophages are required to fully assess these concerns.

The first report describing macropinocytosis as the major endocytic mechanism for ASFV-Ba71V entry in Vero cells was presented by Sánchez et al. [41]. By using field emission electron scanning microscopy (FESEM), it was demonstrated that ASFV triggers cytoplasm membrane perturbation in the form of “ruffles” in Vero cells or “blebs” in IPAM (immortalized porcine alveolar macrophages) cells over the course of internalization. The process triggered by the virus depended on Rac1 Rho-GTPase activation, as inhibition of Rac1 significantly reduced the formation of “ruffles” as well as uptake of the virus. Furthermore, ASFV entry (analyzed for the first time by flow cytometry and confocal laser-scanning microscopy, using a specific monoclonal antibody against the viral capsid protein p72), was shown to be greatly decreased after treatment with the inhibitors of Na^+^/H^+^ channels (EIPA), Pak-1 (IPA-3), PI3K (LY294002), actin cytoskeleton (Cytochalasin D), EGFR (324674), Rac-1 (NSC23766) and tyrosine kinases (Genistein) [41]. As a consequence of the major decrease in viral uptake when cellular factors related to macropinocytosis were impaired, the expression of early and late viral proteins such as p72, p32, p12 and p17, together with viral factory formation and viral production, were also reduced. It was important to demonstrate that the use of inhibitors affecting macropinocytosis inhibited viral entry, without affecting post-entry steps. To achieve this, Sanchez et al. incubated Vero cells with specific inhibitors 60 min before or after virus addition, showing that only PI3K and actin cytoskeleton had a role in both entry and post-entry viral steps, whereas the rest of the cell factors were specifically involved in viral internalization. The authors also demonstrated that ASFV induces uptake of dextran, which is inhibited in the presence of EIPA, finding significant co-localization of viral particles with the fluid phase marker during the first 30 min of infection. In the case of IPAM cells, it was shown that E70-ASFV induces bleb formation during the first 60 min of infection and the viral particles co-localize with the Rock-1 protein. Moreover, viral internalization was inhibited in the presence of blebbistatin, pointing to the role of macropinocytosis also in IPAM cells. These results indicate that both the host cell type and virus isolate are important to define the type of membrane protrusions during ASFV entry by macropinocytosis.

**Figure 2:**
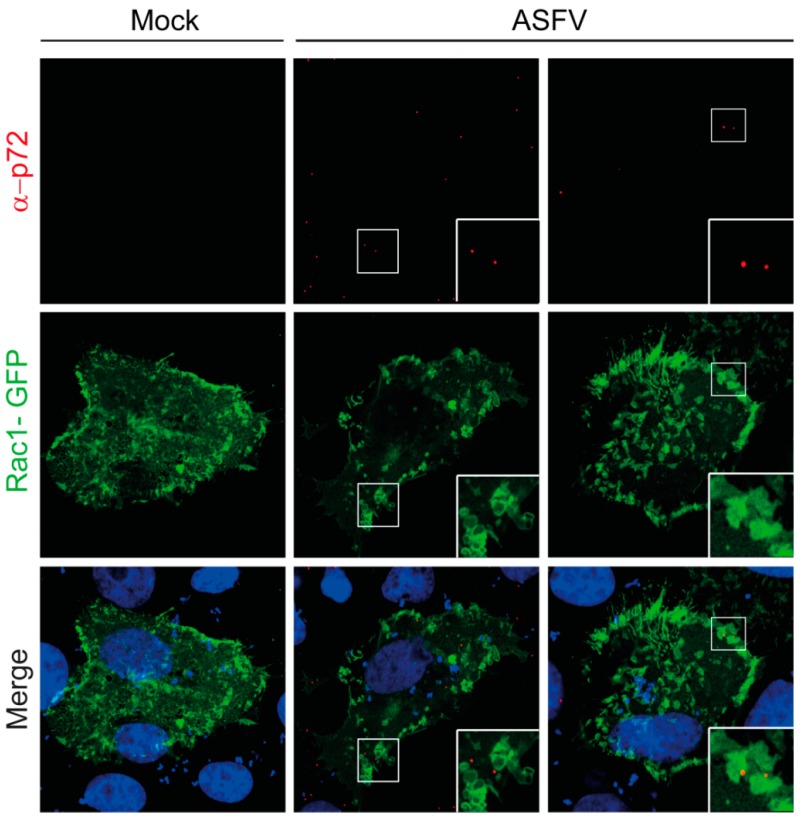
Rac1 localizes to ruffles during ASFV entry. Vero cells were transfected with EGFP-Rac-1 plasmid expression for 16 h and synchronously infected (adsorption period of 90 min at 4 °C) at an multiplicity of infection (MOI) of 10. Cells were fixed and incubated with Topro-3 and monoclonal antibody anti-p72 17LD3 to visualize DNA and viral particles, respectively; samples were processed by CLSM (x63 magnification). Images are representative of three independent experiments (images from D. Pérez-Núñez, E. G. Sanchez and Y. Revilla).

In relation to activation of signaling pathways during ASFV entry, Sánchez et al. showed that ASFV-Ba71V activates cellular factors such as PI3K, Rac-1 GTPase and Pak1 kinase during the first minutes of internalization in Vero cells, focusing on these cell factors as key signals for the induction of macropinocytosis and viral uptake [41]. The virus-induced PI3K activation was measured by analyzing the phosphorylation of Akt, which occurred as early as 10 min after the addition of ASFV to the cells. A similar pattern of activation was found for Pak-1 kinase [41] and Rac-1 Rho-GTPase [41,54] during the first steps of the viral entry. The role of both cellular factors during ASFV infection was also demonstrated by transfecting cells with dominant-negatives for both Pak-1 (pEGFP-Pak-1-AID) and Rac-1 (pcDNA-Rac-1 N17), which inhibited the infection and the synthesis of ASFV early proteins. Moreover, the expression of viral protein p32 was slightly higher in the presence of a constitutive Pak-1 mutant (pEGFP-Pak-1-T423), indicative of a higher degree of infection. Other results from our laboratory have shown that the localization of Rac-1 in ruffles, where viral particles are being uptaken, was also observed during the first minutes of virus internalization, as shown in Figure 2.

**Figure 3:**
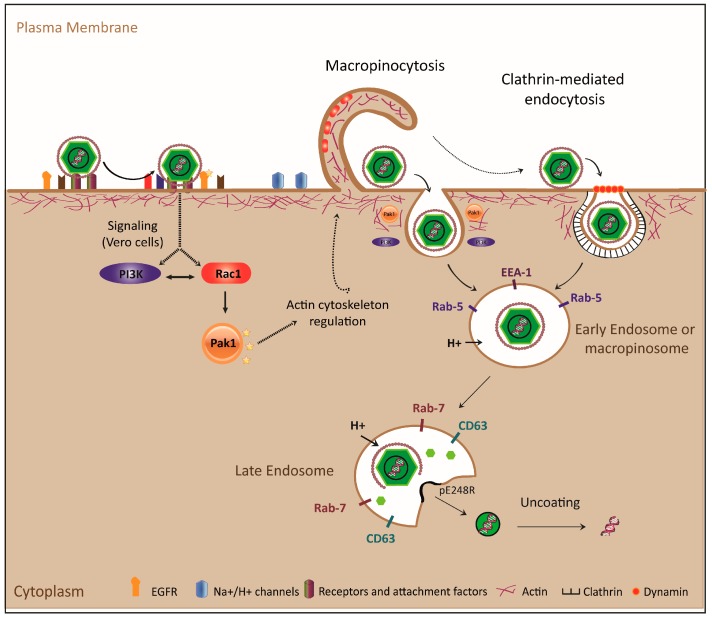
Model for ASFV entry and uncoating. Interaction of the viral particle with membrane receptors and attachment factors activates PI3K, EGFR, Rac1 and Pak-1 signaling pathways, which regulate actin dynamics, forming ruffles to internalize by macropinocytosis in Vero cells. In the case of swine macrophages, although the virus uses macropinocytosis, it does not actively induce the pathway; ASFV is also able to enter cells by CME. After viral uptake, particles are endocytosed in early endosomes or macropinosomes and transported to late endosomes where the pH-dependent uncoating process takes place. The viral outer envelope is disassembled and the inner envelope fuses with the endosomal membrane, delivering viral cores to cytosol, where viral protein pE248R plays an important role.

The mechanism of macropinocytosis induction by ASFV in Vero cells has been controversial. Hernaez et al. [39] reported that the virus does not induce membrane perturbation nor Pak-1 phosphorylation during the entry process into PAM or Vero cells, in contradiction to what Sanchez et al. demonstrated [41]. It has been speculated that the observed differences could lie in the different viral preparations used in the two studies, as Hernaez et al. used virus purified by Percoll, and others including Sanchez et al. [20,40,42] used non-purified virus. Although the use of purified virus may be convenient for certain types of studies, other experimental approaches indicate that it can affect the envelope of the virus, resulting in a heterogeneous mixture of viruses with or without an external membrane. Moreover, purification has been shown to not be crucial to Vaccinia virus (VV) entry, as both purified and non-purified viruses were able to induce macropinocytosis [55,56]. Hernaez et al. claimed that the cellular debris in viral preparations could be responsible for activating macropinocytosis in Vero cells [39], but they did not demonstrate that cellular debris alone could induce membrane perturbations. Besides, it has been demonstrated that ruffling is a transitory event in Vero cells, with a maximum level of ruffle formation at 10 min post-infection and its almost complete disappearance after 60 min, correlating with the viral internalization [41]; if this process was induced by cellular debris present in the viral preparation, the level of ruffling would be expected to remain constant as long as the cellular debris was present, independently of the viral entry kinetic. On the other hand, as macropinocytosis is not constitutively activated in Vero cells, it is difficult to think that the virus would enter by macropinocytosis if the pathway was not activated by the virus itself. Not only that, but also since Vero cells are successfully infected by purified and non-purified virus, if the entry of non-purified viruses by macropinocytosis were only a consequence of activation by cellular debris, it would not be compromised in the presence of different pharmacological inhibitors as previously published [41]. In this regard, unpublished data from our lab shows that the uptake of non-purified vs. Percoll-purified ASFV-Ba71V is inhibited in the presence of EIPA, suggesting that this pathway is key for virus entry, and therefore, is activated by the virus in Vero cells. Finally, alternative mechanisms such as phagocytosis have been suggested to be used by ASFV to enter primary macrophages [57]. Figure 3 and Table 1 show the endocytic mechanisms used by ASFV to enter host cells, and the cellular factors needed for its internalization.

The currently-published works reveal the versatility of ASFV to infect both Vero and PAM by means of at least two different routes, CME [39,40,42,58] and macropinocytosis [39,41], which rather than being exclusive processes, seem to be cooperative, since actin rearrangement during ASFV macropinocytosis could contribute the clathrin-mediated viral endocytosis (Figure 3).

## 2. The ASFV Endosomal Pathway

### 2.1. ASFV Movement through the Endolysosomal System

#### 2.1.1. ASFV Movement across the Endolysosomal System

After internalization, ASFV traffics throughout the entire endolysosomal system. A number of studies have been carried out analyzing the location of ASFV at different times post-entry, by using confocal [20,39] or electron microscopy [39], as well as in situ fluorescent microscopy [39]. ASFV has been detected by immunofluorescence after the staining of different viral proteins, including the capsid (p72 and pE120R), core (p150) [20], or the p17 internal envelope protein [39].

Shortly after infection (5–30 mpi), capsid and inner envelope proteins are detected in early endosomes or macropinosomes, colocalizing with specific markers such as EEA1 and Rab5 [39]. At later times (30–90 mpi), virions co-localize with markers of late endosomes and lysosomes such as CD63 (marker for MVB), Rab7 (late endosome) or Lamp 1 (lysosomes), shown mainly by way of the envelope protein (p17) or core (p150) [20], since the virion is already uncapped by that time. Virions are not detected in lysosomes by immunofluorescence, although they have been observed by electron microscopy [39]. These results have been further confirmed by electron microscopy of immunogold-labeled cryosections using antibodies against components of different compartments, such as transferrin receptor (early endosome), CD63 (MVB), Lamp 1 and cathepsin L (lysosomes) [39]. Another approach to observe intracellular virus trafficking after internalization has been in situ fluorescence using DiD-labeled viral particles. The assay was performed on COS cells transfected with GFP-Rab5 or GFP-Rab7, and ASFV was observed in Rab5+ vesicles at early times, and in Rab7 + vesicles at late times [39]. 

In addition to Vero and COS cells, PAM have also been used to analyze cellular structures during infection. Morphological criteria such as vesicle size, presence of intraluminal vesicles and membrane sheets (compatible with late endosomes) have been used since no appropriate porcine markers are available for specific compartment identification at the microscopic level [39]. These studies have also revealed the importance of viral transit through the endolysosomal system for correct uncoating and the subsequent stages of the viral life cycle. In particular, the key role of Rab7, an important regulator of the maturation of early to late endosomes, has been demonstrated. On the one hand, Rab7 negative expression (DN) has been shown to prevent the progression of infection in Vero cells, as only a negligible number of transfected cells were infected compared to empty vector controls [20]. On the other hand, it has been shown that partial silencing of Rab7 decreases the expression of ASFV early protein p32 in COS cells, also suggesting that there would be inhibition in the progression of ASFV through the endolysosomal route, therefore giving Rab7 an important role in this process [39].

#### 2.1.2. Importance of Acidification and Other Factors (i.e., Lipid Composition) in the Progression of ASFV through the Endolysosomal Route

Productive ASFV infection depends on the maturation of endosomes, a process involving morphological, locational and biochemical changes, such as vesicle acidification, as the cycle progresses from early to late endosomes. A direct relationship has been established between this maturation process of the endosomal pathway and the ability of ASFV to progress in the cell [20,39]. In this regard, one of the earliest key aspects is the acidification of endosomes. There is a gradation in pH between the vesicles of the endolysosomal system, which progressively acidify from the early endosomes with a pH near 7 through the late endosomes and lysosomes whose pH is around 5. Such acidification is not only necessary for the progression of the endolysosomal cycle, but also for ASFV to be able to complete its infectious cycle. Indeed, using bafilomycin A1 (Baf A1), which blocks the H^+^ ATPase pump by preventing acidification of the endosomes, also prevented the virus from expressing early protein p32 [20]. This inhibition only occurred when the drug was added before viral adsorption, and not when it was added 3 h later (post-internalization), indicating that the drug is only effective during the first steps of internalization/decapsulation. This has also been confirmed by assessing the expression of p32 in macrophages in a drug-dependent manner [39]. Other drugs that affect the physiology of the endosomal system also prevent the progression of ASFV infection, including nocodazole, which depolymerizes microtubules, thus preventing the movement of early endosomes [39]. 

Finally, it is likely that the correct metabolism of phospholipids from endosomal membranes may also play a role in the progression of ASFV. Prior to virus internalization, specific inhibitors of enzymes involved in the metabolism of such phospholipids, including the PI3-kinase inhibitor wortmannin or LY294002 and PIKfyve inhibitor YM201636, inhibit viral infection, as observed in the decreased expression of early protein p32 and late proteins p72 and p24 [20,41].

### 2.2. ASFV Uncoating

ASFV uncoating involves the loss of the outer layers of the virion (outer membrane and protein capsid) in multivesicular late endosomes, and the later fusion of the inner membrane with the endosomes, releasing the nude core to the cytosol. The viral uncoating and release of cytoplasmic cores from the endosomes begins at 60 mpi, and is also dependent on vacuolar acidification, being inhibited by lysosomotropic drugs [3]. In a recent study using electron microscopy [39], viral particles were localized to the different endosomal compartments, which, together with the layers displayed by the virion, allowed the definition of maturation steps and the degree of uncoating. Most of the particles found in the early endocytic vesicles had the structure of intact virions, whereas the majority (more than 85%) of particles within the late (multivesicular) endosomes had lost their capsid proteins, and a large proportion (more than 50%) had also lost their outer membrane. In addition, in most cases where the outer membrane was still present, it appeared broken and dissociated.

Once the outer layers of the virion have undergone degradation, the internal membrane of the virion is exposed within multivesicular endosomes. In fact, many of these virions are anchored to the membranes of these endosomes, as observed by electron microscopy. Viral particles whose inner membrane appeared fused to the membrane of the vesicular endosomes were also observed, suggesting that this is the process by which the naked core is released to the cytosol. In fact, such cores are fundamentally located in the cytosol in direct contact or close proximity to the membrane of vesicular endosomes. It seems that ASFV uses the internal membrane to release cores containing the viral genome to the cytosol by a process of lipid fusion [39]. It is noteworthy that, in addition to viral progression through the endolysosomal cycle, the disassembly and uncoating of the viral particles is an acid-pH-dependent process.

Treatment with Baf A1, which inhibits the acidification of endosomes, prevents the progression of the viral particle through the endosomal route, being mostly blocked within small vesicles (<200 nm) in porcine macrophages due to a general blockage of endosome maturation [39]. In addition, the particles found in these endosomes appear mostly intact, in agreement with the absence of cores in the cytosol; this indicates not only a blockage in viral progression but also a blockage in the decapsidation of the virion. In fact, the inhibition of acidification by Baf A1 allows the virion to be visualized in late Rab7 + endosomes using antibodies against the p72 capsid protein [20]. This indicates that, unlike what occurs during productive infection, uncoating has not occurred in these compartments due to lack of acidic pH. A direct effect of pH acidification on the integrity of extracellular virions has been visualized by electron microscopy of in vitro purified particles [39]. After exposure to different buffers with pH values ranging from 4.0–8.0, virions remained intact at pH > 5.0, but showed clear signs of disassembly and loss of structure below pH 5.0. Accordingly, immunogold staining showed that both capsid (p72) and inner membrane (p17) proteins were accessible to specific antibodies, whereas antibodies were not able to penetrate intact particles. Therefore, disassembly of the capsid and dissociation of the outer membrane are processes that are strongly dependent on acidic pH.

In addition to pH, it appears that certain viral envelope proteins play a key role in virion disassembly. Core detachment from the inner membrane may be a key process for core release to the cytoplasm, since the core of incoming particles is clearly detached in contrast to what happens during ASFV morphogenesis. This attachment depends on viral protein pp220, a polyprotein precursor of four major components of the core [60]. When the proteolytic process of pp220 occurs, the mature core separates from the inner membrane, whereas core detachment was not detected when proteolysis was impaired [61]. Hence, these proteolytic events, linked to ASFV maturation, seem to be important for core release. Another important factor is the ASFV protein pE248R, an internal envelope protein similar to the Vaccinia L1 protein, which is part of the multiprotein entry and fusion complex [62]. Its function was first studied using an inducible mutant under the IPTG promoter [63], which demonstrated that mutants lacking this protein were viable and able to form viral particles morphologically similar to wild type virions. However, the defective particles were unable to carry out productive infection, with blockage at a post-entry step. A recent study determined that in fact this protein would be involved in the fusion of the inner membrane with the membranes of multivesicular endosomes [39]. Under permissive or restrictive conditions, its distribution among the endosomes was found to be similar to wild type virions, but with a drastic decrease in the appearance of cores in the cytosol. Viral particles mostly accumulated in vesicles compatible with multivesicular lysosomes and lysosomes, as observed by electron microscopy. It follows that pE248R is required for fusion between internal and endosomal membranes and therefore for core release, but not for the disassembly of the external layers of the virion. 

Figure 3 and Table 1 schematically show the endocytic mechanisms used by ASFV to enter into host cells and the cellular factors required for viral internalization, endosomal trafficking and uncoating.

## 3. Conclusions

ASFV dissemination from Sardinia and Caucasian areas to EU countries has recently appeared, and current outbreaks have been declared in Estonia, Lithuania, Latvia, Poland and recently the Czech Republic, thus making the expansion of knowledge and tools for protection against this virus urgent. ASFV infection is characterized by an absence of neutralizing immune response, which has so far impaired the development of a conventional vaccine. Knowledge of the viral and cellular factors involved in entry/internalization and endosomal trafficking can help to develop new potential therapies against the virus. Recombinant viruses lacking proteins that trigger viral decapsidation or the inhibition of early signaling pathways may be useful as candidates for future live-attenuated vaccines for ASF.

## Figures and Tables

**Table 1:** Cellular factors involved in ASFV entry. The table shows the cellular factors that play an important role during internalization through the plasma membrane (viral uptake) and/or post-internalization steps (endosome transport) according to published data. The type of inhibitor or dominant negative construct used for analysis is also shown.

Cellular Factors	Inhibitors	Internalization (Viral Uptake)	Post-Internalization (Endosome Transport)
Na^+^/H^+^ channels	EIPA	Yes [39,41,42]	Yes [42]/No [40,41]
Actin	Cytochalasin D and B Latrunculin A	Yes [39,41,42]/No [21]	Yes [41,42,57]
Myosin II	Blebbistatin	Yes [41]	Yes [41]
EGFR	324674	Yes [41]	Unknown
PI3K	LY294002Worthmanin	Yes [41,42]	Yes [20,39,40,41]
Rac1	NSC23766, Rac1-N17	Yes [41]/No [40]	Yes [40,41]
Pak1	IPA-3, Pak1-AID	Yes [39,41]	No [41]
Tyrosin kinases	Genistein	Yes [41]	Unknown
Dynamin-2	Dynasore	Yes [39,41,42]	Yes [40,41,42]
Clathrin	Clorpromazine	No [41]	Yes [40,41]
Microtubules	Nocodazol	No [41]	Yes [39,41,59]
Vacuolar acidification	Cloroquine, NH_4_Cl, Bafilomycin A	No [2,3]/Yes [42]	Yes [20,39,41,57]
Cholesterol	MβCD	No [22]	Yes [20,22]
Rab-7	Rab-7-T22N Rab-7 siRNA	Unknown	Yes [10,11]
